# Probabilistic seismic demand assessment of special truss moment frames with Vierendeel panels under geometric variations

**DOI:** 10.1038/s41598-026-42239-y

**Published:** 2026-03-19

**Authors:** Aliakbar Yahyaabadi, Mohsen Gholami, Sadegh Garivani

**Affiliations:** Faculty of Engineering, University of Bojnord, Bojnord, Iran

**Keywords:** Special truss moment frame (STMF), Vierendeel truss panel, Incremental dynamic analysis (IDA), Probabilistic seismic demand analysis (PSDA), Collapse fragility curve, Seismic demand prediction equation, Engineering, Solid Earth sciences

## Abstract

Special truss moment frames (STMFs) have attracted growing interest due to the demand for column spacing that exceeds conventional restrictions. This study investigates the seismic performance of 27 three-span STMFs with mid-span Vierendeel truss panels having different structural configurations. The considered STMFs include three, six, and nine stories; span lengths of 10, 15, and 20 m; and have special ductile segment lengths of 1, 1.5, and 2.25 m. Incremental dynamic analyses (IDA) of STMFs were performed under 22 strong ground motions to assess the dependence of structural responses on geometric configurations while explicitly accounting for record-to-record uncertainty. Seismic demand models and fragility curves were constructed using Bayesian statistics and combined with site-specific hazard curves in a probabilistic seismic demand analysis (PSDA). The results indicate that the structural seismic performance of STMFs is strongly affected by the height-to-span (h/L) and the special segment length-to-span (Lₛ/L) ratios. Seismic demand predictive equations in terms of geometric parameters were proposed and validated against numerical data, confirming their reliability for preliminary seismic assessment. The results demonstrate that, within the studied range of parameters, reducing either the height-to-span ratio (h/L) or normalized special segment length (Lₛ/L) leads to improved seismic performance of STMFs.

## Introduction

Multi-story buildings with column spacing greater than conventional limits have received increased attention lately. Moment-resisting frames with truss beams are one of the most effective and widely used structural systems for this purpose. Truss beams, carrying vertical loads and resisting lateral forces, are economical and have simplified connections to columns. Additionally, the open web configuration in trusses can be used for the installation of mechanical and utility systems.

Goal and Itani examined the seismic performance of truss moment-resisting frames by performing a full-scale experimental study^1^. They observed a notable reduction in initial stiffness and lateral strength under cyclic loading. In these systems, truss beams have higher stiffness and strength in contrast to the fundamental design philosophy of strong column–weak beam. As a result, plastic hinges often form in the columns, leading to reduced overall ductility and the potential for undesirable failure modes during severe seismic events; therefore, truss configurations should be modified to address this issue. In the mid-span region of the truss, where gravity-induced forces are minimal, diagonal web members can be removed (forming a Vierendeel-type panel) or replaced with reduced-strength members. This local reduction in strength helps create plastic hinges in a specific area of the truss and therefore increase energy dissipation capacity. This inelastic region, referred to as the special segment, forms the basis of the special truss moment frame (STMF) system. The expected plastic hinge locations are at the ends of the horizontal members within this segment^2^.

Chao and Goal looked into the performance-based plastic design (PBPD) method for STMFs and found that their approach successfully met the design goals, such as reaching the target interstory drift and creating the intended plastic hinge mechanism^3^. Asghari et al. demonstrated that using STMFs with Vierendeel special segments is efficient and economical for long-span structures in seismic-prone regions^4^. Kim and Park studied how different designs of STMFs, which vary in length, number of stories, and special segment lengths, respond during a progressive collapse using nonlinear static analysis. They demonstrated that additional research is necessary to evaluate the seismic performance of these frames under severe earthquakes^2^. In a different study, a comparative study was conducted by Gade and Sahoo to estimate the collapse capacity of a nine-story STMF using ASCE 7 guidelines and the PBPD methodology^5,6^. The comparison revealed that the PBPD approach yielded superior collapse performance at FEMA P-695 seismic hazard levels^7^.

Sophianopoulos and Ntina showed that using buckling-restrained braces in one-story single-span STMFs makes them lighter and causes less peak displacement^8^. Researchers have recently looked into multi-story, multi-bay STMFs that use X-diagonal shape memory alloy (SMA) bars. They found that designing the SMA bars based on the maximum expected vertical shear strength led to more uniform inter-story drift patterns and lower drift demands on the upper floors compared with a conventional STMF configuration^9^. Some studies looked into the benefits of hybrid damping systems in STMFs. For example, using buckling-restrained braces with viscous and friction dampers in STMFs led to more energy absorption and less displacement under far- and near-field earthquakes^10^.

Probabilistic seismic demand analysis (PSDA) can be used as a powerful tool for estimating seismic response that accounts for uncertainties in both seismic demand and seismic hazard analyses^11^. Some studies have included ground motion uncertainty in the design of nonlinear truss-like structures by using adaptive optimization methods to get the desired member ductility distributions^12^. Recent developments have also applied probabilistic seismic assessment to long-span structural systems. Chen et al. conducted a PSDA of a mid-rise large-span cassette structure under 25 FEMA motions, taking into account directional uncertainty. In conducting PSDA, they used suitable intensity measures developed based on 110-record screening^13^. Abdollahzadeh et al. used the capacity spectrum method to generate fragility curves for special truss moment frames^14^. Kumar et al. developed fragility curves for various performance levels for a nine-story STMF with a special Vierendeel zone^15^. Kumar and Sahoo extended this work by analyzing STMFs of different heights and special panel configurations using a suite of strong ground motion records. They discovered that taller buildings show better collapse resistance with a single-panel Vierendeel truss, while shorter buildings benefit from multiple Vierendeel panels^16^. Some studies have developed prediction equations to estimate seismic demands of steel structures. For example, Diamantis et al. proposed relationships between physical properties of viscous dampers and equivalent damping ratios. Their study shows that simplified predictive methods can help assess seismic response without explicit damper modeling^17^. These approaches emphasize that uncertainty modeling for seismic performance evaluation is getting increasing attention. A few studies have established STMF fragility curves, but probabilistic seismic demand evaluations remain limited.

To address this gap, incremental dynamic analyses (IDA) of 27 special truss moment frames (STMFs) were conducted under 22 strong ground motion records in the present study. The models represent a wide range of geometric parameters, including three, six, and nine stories with a story height of 6 m, span lengths of 10, 15, and 20 m, and Vierendeel special segment lengths of 1, 1.5, and 2.25 m. Seismic performance and collapse risk of the STMFs were evaluated using a probabilistic seismic assessment framework. Additionally, prediction equations were developed for seismic demands, collapse fragility curves, and collapse probabilities in terms of STMFs’ geometric parameters. This paper facilitates the primary assessment of STMFs and enhances current knowledge by correlating structural geometric features with the seismic performance of special truss moment frames.

## General specifications, design, and modeling of the frames

In this study, truss moment frames having special Vierendeel truss segments with various configurations were investigated. The special truss moment frames (STMFs) examined have three-span configurations and were designed with 3, 6, and 9 stories; spans measuring 10, 15, and 20 m in length; and special zones measuring 1, 1.5, and 2.25 m in length. All floors were assumed to have the same height of 6 m, comprising the 4.5 m of clear floor height and 1.5 m of truss depth; additionally, the load-bearing width of the frames was assumed to be 10 m. The dead and live loads on each floor were considered as 500 kg/m^2^. This dead load is in addition to the self-weight of the structural steel members, which is considered separately. Additionally, the roof live load and snow load were taken as 150 kg/m^2^.

**Fig. 1:**
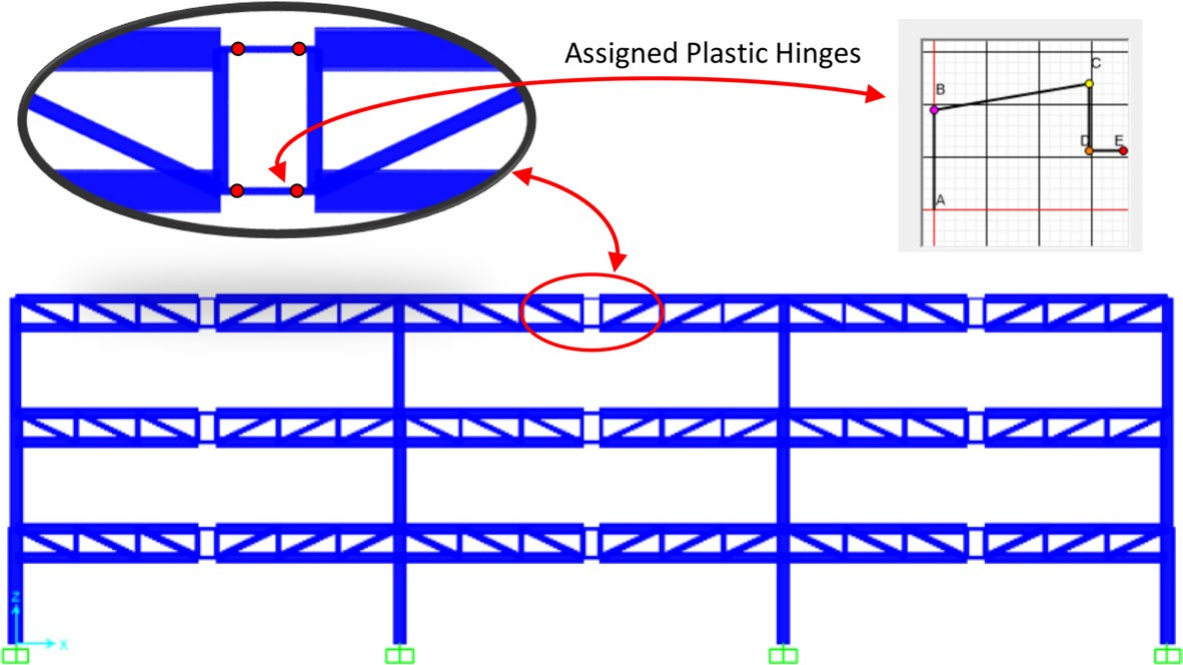
Side view of the S3-L20-Z1 STMF, showing plastic hinges at the ends of the special truss segments. Hinges in columns and truss members outside the special segments are also defined but not shown for clarity.

Figure 1 shows the side view of one of the STMF frames considered in this study, illustrating the overall frame geometry and the position of the special Vierendeel truss segment. Three different options for the structure height, span length, and special zone length were investigated to see how each of these sizes affects seismic performance. The frames were designed according to the AISC360-16^18^ and in compliance with the seismic provisions of special truss moment frames (Section E4) of the AISC341-16^19^. Considering the truss depth h_T_ = 1.50 m, special segment lengths (L_s_ = 1.00 m, 1.50 m, and 2.25 m) were selected in a way that, according to AISC 341 − 16, Ls/h_T_ ratios fall in that range of 0.67 and 1.5.

The format STMF Sx–Ly–Zz was used to name different special truss moment frames (STMFs) to ensure simple referencing: Sx indicates the number of stories (S), Ly denotes the span length in meters (L), and Zz represents the length of the special segment (Vierendeel zone) in meters (Z). For example, STMF S3–L15–Z1 refers to a 3-story frame with a 15-meter span length and a 1-meter special segment. This notation is used throughout the text, tables, and figures to differentiate between the 27 STMF configurations studied.

The models are assumed to be located in a high-seismic region with a design peak ground acceleration of 0.30 g (Bojnord, Iran). The site soil is classified as very dense, with an average shear wave velocity, ${\stackrel{-}{V}}_{s,30}$, in the range of 360 to 760 m/s. The structural models are assumed to represent a large department store, so an importance factor of I = 1.2 was used in the design. The frame behavior factor was set to *R* = 7, and for the design of the frame members, the target demand-to-capacity ratio for the majority of members was maintained between 0.7 and 1.0.

The connection between the upper and lower chords of the truss beam and the columns is rigid, and the connection of the diagonal members of the truss to the upper and lower chords is simple. Rigid diaphragms were assigned to the floors. The steel used in the structure was ST37, with yield and ultimate strengths of 240 and 370 MPa, respectively. Columns were designed to have sufficient resistance to the axial forces resulting from the amplified seismic load combinations, according to AISC 341 − 16^19^. In addition, truss beam members were designed to confine nonlinear ductile behavior to a designated special zone, while the members outside this area remained elastic under gravity and lateral loads. For this purpose, the overstrength factor Ω_o_ = 3 was used to amplify the earthquake loads in the design combinations for members outside the special zone^19^.

The STMFs were designed based on the results of a linear static analysis, considering P-delta effects. The maximum relative drift of the stories was limited to 0.02h_sx_ (where h_sx_ is the story height). The formation sequence of plastic hinges was controlled using nonlinear static analyses (push-over) to ensure that plastic joints formed first in the special Vierendeel segment of the beam, then in other truss beam members, and finally in the columns, while also ensuring that the plastic hinges were uniformly distributed along the height of the frames. The columns’ nonlinear behavior was modeled using shear and axial-flexural plastic hinges, while the truss beam members were modeled using axial-flexural plastic hinges. The lateral load pattern in the nonlinear static analysis was defined using two distributions: one proportional to the lateral forces obtained from the dynamic analysis (considering the number of modes that contribute 90% of the structural mass) and another based on a uniform distribution proportional to the floor weights^20^.

Table 1 presents the main section dimensions of three sample frames (S3-L10-Z1, S6-L10-Z1, and S9-L10-Z6). Modal periods, effective modal mass ratios, and design base shear values of the STMF models are summarized in Table 2. As shown in this table, the first-mode (fundamental) period of the studied frames varies from 0.8 to 3.2 s, reflecting differences in the height and lateral stiffness of the structures.

**Table 1:** Section dimensions (mm) of representative STMF frames, all sections are hollow rectangular tubes.

Structure ID	Story	Columns	Chord members	Vertical members	Diagonal members	Special chord members
3 S-L10-Z1	1	400 × 400 × 40	220 × 220 × 25	120 × 120 × 12.5	120 × 120 × 12.5	140 × 140 × 14.2
	2	360 × 360 × 35	200 × 200 × 25	100 × 100 × 12.5	100 × 100 × 12.5	120 × 120 × 12.5
	3	300 × 300 × 30	200 × 200 × 16	100 × 100 × 8	100 × 100 × 8	80 × 80 × 8
6 S-L10-Z1	1	4× Tube 300 × 300 × 30 @ 30 (cm)	240 × 240 × 20	100 × 100 × 12.5	100 × 100 × 12.5	140 × 140 × 14.2
	2	4× Tube 300 × 300 × 20 @ 30 (cm)	240 × 240 × 22.2	100 × 100 × 12.5	100 × 100 × 12.5	140 × 140 × 14.2
	3	4× Tube 300 × 300 × 16 @ 30 (cm)	240 × 240 × 22.2	100 × 100 × 12.5	100 × 100 × 12.5	140 × 140 × 14.2
	4	400 × 400 × 40	300 × 300 × 30	100 × 100 × 10	100 × 100 × 10	140 × 140 × 14.2
	5	380 × 380 × 35	280 × 280 × 30	100 × 100 × 10	100 × 100 × 10	140 × 140 × 14.2
	6	300 × 300 × 28	200 × 200 × 20	80 × 80 × 8	80 × 80 × 8	100 × 100 × 10
9 S-L10-Z1	1	4× Tube 300 × 300 × 40 @ 50 (cm)	300 × 300 × 30	120 × 120 × 12.5	120 × 120 × 12.5	140 × 140 × 14.2
	2	4× Tube 300 × 300 × 30 @ 40 (cm)	300 × 300 × 30	120 × 120 × 10	120 × 120 × 10	140 × 140 × 14.2
	3	4× Tube 300 × 300 × 30 @ 40 (cm)	300 × 300 × 30	120 × 120 × 10	120 × 120 × 10	140 × 140 × 14.2
	4	4× Tube 300 × 300 × 25 @ 40 (cm)	300 × 300 × 30	100 × 100 × 12.5	100 × 100 × 12.5	140 × 140 × 12.5
	5	4× Tube 300 × 300 × 30 @ 30 (cm)	300 × 300 × 30	100 × 100 × 12.5	100 × 100 × 12.5	120 × 120 × 12.5
	6	4× Tube 300 × 300 × 20 @ 30 (cm)	300 × 300 × 30	90 × 90 × 8	90 × 90 × 8	120 × 120 × 12.5
	7	4× Tube 300 × 300 × 16 @ 30 (cm)	300 × 300 × 30	80 × 80 × 8	80 × 80 × 8	100 × 100 × 12.5
	8	400 × 400 × 35	300 × 300 × 30	80 × 80 × 8	80 × 80 × 8	100 × 100 × 10
	9	320 × 320 × 30	260 × 260 × 25	70 × 70 × 5	70 × 70 × 5	80 × 80 × 8

**Table 2:** Fundamental dynamic properties and design base shear of the analyzed STMF structures.

Structure ID	T_1_ (sec)	T_2_ (sec)	Effective modal mass (Mode 1)	Effective modal mass (Mode 2)	Design base shear (KN)
S3-L10-Z1	1.034	0.435	0.776	0.146	583.7
S3-L10-Z1.5	1.141	0.490	0.749	0.158	582.7
S3-L10-Z2.25	1.311	0.513	0.737	0.142	584.7
S3-L15-Z1	0.960	0.397	0.747	0.162	903.5
S3-L15-Z1.5	1.086	0.473	0.717	0.143	901.5
S3-L15-Z2.25	1.419	0.676	0.651	0.136	897.6
S3-L20-Z1	0.750	0.447	0.705	0.127	1484.3
S3-L20-Z1.5	1.026	0.627	0.513	0.272	1475.4
S3-L20-Z2.25	1.178	0.690	0.566	0.191	1465.6
S6-L10-Z1	1.576	0.619	0.776	0.096	652.4
S6-L10-Z1.5	1.708	0.664	0.775	0.097	648.4
S6-L10-Z2.25	1.818	0.693	0.772	0.099	650.4
S6-L15-Z1	1.292	0.508	0.754	0.114	1322.4
S6-L15-Z1.5	1.362	0.544	0.748	0.116	1305.7
S6-L15-Z2.25	1.464	0.576	0.749	0.112	1299.8
S6-L20-Z1	1.270	0.490	0.756	0.113	1962.0
S6-L20-Z1.5	1.289	0.494	0.757	0.112	1953.2
S6-L20-Z2.25	1.335	0.508	0.758	0.109	1939.4
S9-L10-Z1	2.177	0.872	0.711	0.132	1064.4
S9-L10-Z1.5	2.526	1.064	0.692	0.142	1057.5
S9-L10-Z2.25	3.152	1.195	0.716	0.122	1052.6
S9-L15-Z1	1.721	0.706	0.730	0.112	1636.3
S9-L15-Z1.5	2.068	0.835	0.735	0.110	1721.7
S9-L15-Z2.25	2.717	1.149	0.713	0.108	1706.0
S9-L20-Z1	1.879	0.719	0.738	0.110	2485.9
S9-L20-Z1.5	2.151	0.846	0.718	0.110	2470.2
S9-L20-Z2.25	2.490	0.964	0.707	0.111	2450.5

In this study, SAP2000 v17 software was used to perform all incremental dynamic analyses required for conducting PSDA^21^. Figure 1 shows the S3‑L10‑Z1 special truss moment frame, highlighting the concentrated plastic hinges in the special truss beam segments. Because these beams have seismically compact sections, the hinges in the special regions were defined to provide maximum ductility according to seismic compactness requirements. Plastic hinges were also defined for columns and truss members outside the special regions, but they are not shown in the figure to avoid clutter. The columns’ nonlinear behavior was modeled using shear and axial-flexural plastic hinges, while the truss beam members were modeled using axial-flexural plastic hinges. Truss diagonal members were assigned axial hinges to capture tensile and compressive yielding. The modeling parameters for the hinge properties and force–deformation relationships were defined according to ASCE/SEI 41‑17^22^. The unloading and reloading behavior under cyclic seismic loading was modeled using the kinematic hardening hysteresis method. Structural damping was considered using 5% Rayleigh damping, calibrated based on the first mode and the mode at which cumulative mass participation exceeds 95%.

It should be mentioned that most members outside the special regions remain largely elastic under seismic loads, following the design philosophy of AISC 341‑16 for these frames. This concentrates the nonlinear response in the special truss segments, which are designed to accommodate concentrated plasticity and provide sufficient ductility. Therefore, the frames can exhibit significant inelastic behavior under seismic demands.

## Incremental dynamic analysis of STMFs

Incremental dynamic analysis (IDA) is a set of nonlinear dynamic analyses in which a structure is subjected to a ground motion record that is incrementally scaled to different levels of intensity using a selected intensity measure^23^. This method has been extensively employed in recent studies to establish the relationship between intensity measures and structural demand or collapse capacity for probabilistic seismic demand prediction^24^.

In this study, 22 earthquake records recommended by the FEMA P695 guidelines were used to perform the analyses^7^. The records were obtained from 14 far-field earthquake events between 1971 and 1999. Rupture distances range from 11.1 km to 26.4 km, with an average rupture distance of 16.4 km, and the earthquake magnitudes range from 6.5 to 7.6, with an average magnitude of 7.0. Far-field records were selected because they provide a representative scenario for most urban areas where the nearest active faults are generally located more than 10 km away. For Bojnord, this choice is particularly relevant when combining the PSHA results with IDA to estimate collapse probability, as the city’s center is more than 10 km from the nearest active fault, while some outskirts may be closer. Outside this specific application to Bojnord, the far-field records ensure generality and can be applied to other regions with similar fault distances. Future studies could examine near-field records to evaluate potential pulse effects for areas located less than 10 km from active faults.

The pseudo-spectral acceleration at the first-mode period, S_*a*_(T_1_), was chosen to scale the records because it is a standard measure used in many studies of incremental dynamic analysis, and seismic hazard curves are also available for this measure in different earthquake-prone areas. The maximum inter-story drift ratio (maximum during the earthquake duration and among all stories), represented by *IDR*_max_, was used as the seismic demand parameter, as it effectively predicts both structural and non-structural damage^25^.

**Fig. 2:**
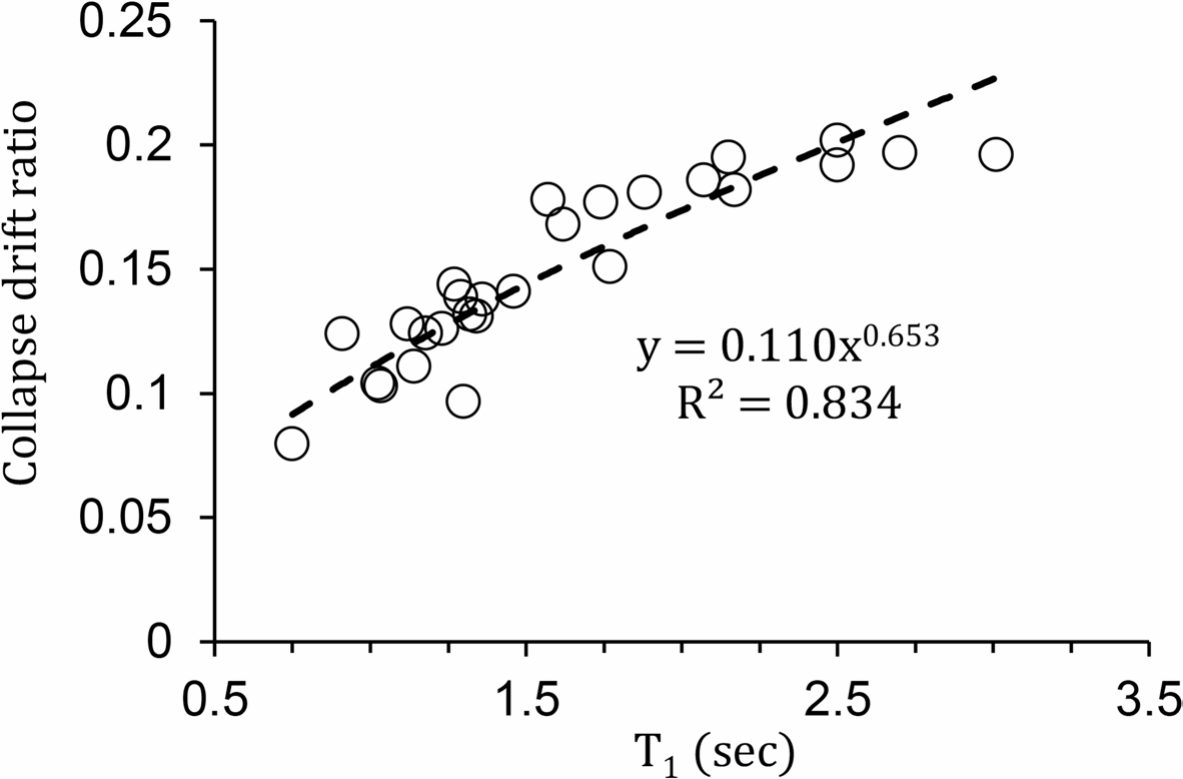
Collapse drift ratio prediction as a function of the first-mode period for the 27 special truss moment frames analyzed in this study.

Incremental dynamic analysis was performed for each of the 27 frames under 22 ground motion records. The collapse point was defined as a decrease in the slope of the IDA curve by more than 20% relative to the initial slope, or as numerical instability in the dynamic analysis^23^. Figure 2 presents the mean collapse drift ratio, $\overline {{IDR}}$_max, c_, for each frame. As can be seen, $\overline {{IDR}}$_max, c_ varies among frames from about 0.075 to 0.200, illustrating differences in structural response. The relationship between $\overline {{IDR}}$_max, c_ and the first-mode period is highlighted as a representative observation from the IDA results.

## Probabilistic seismic demand analysis using the Bayesian statistical method

Seismic demand prediction is one of the most important components of modern performance-based seismic design. The most significant challenge in estimating seismic demand is the presence of numerous uncertainties and inherent randomness. These uncertainties can be categorized into two groups: those associated with ground motions (such as earthquake magnitude, distance, etc.) and those related to the nonlinear behavior of the structure (such as stiffness, ductility, and nonlinear performance characteristics). Probabilistic seismic demand analysis (PSDA) is employed to account for this randomness and uncertainty in seismic demand estimation^26^.

Probabilistic seismic demand analysis is an appropriate approach to calculate the annual probability of exceeding a specified seismic demand parameter for a structure at a given site. This method is based on categorizing the results from nonlinear dynamic analyses of the structure under different ground motion records into two groups: non-collapse data and collapse data. The total theory of probability then combines these results with the findings from the probabilistic seismic hazard analysis (PSHA) for the location^26^.

### Development of the seismic demand model for non-collapse data

**Fig. 3:**
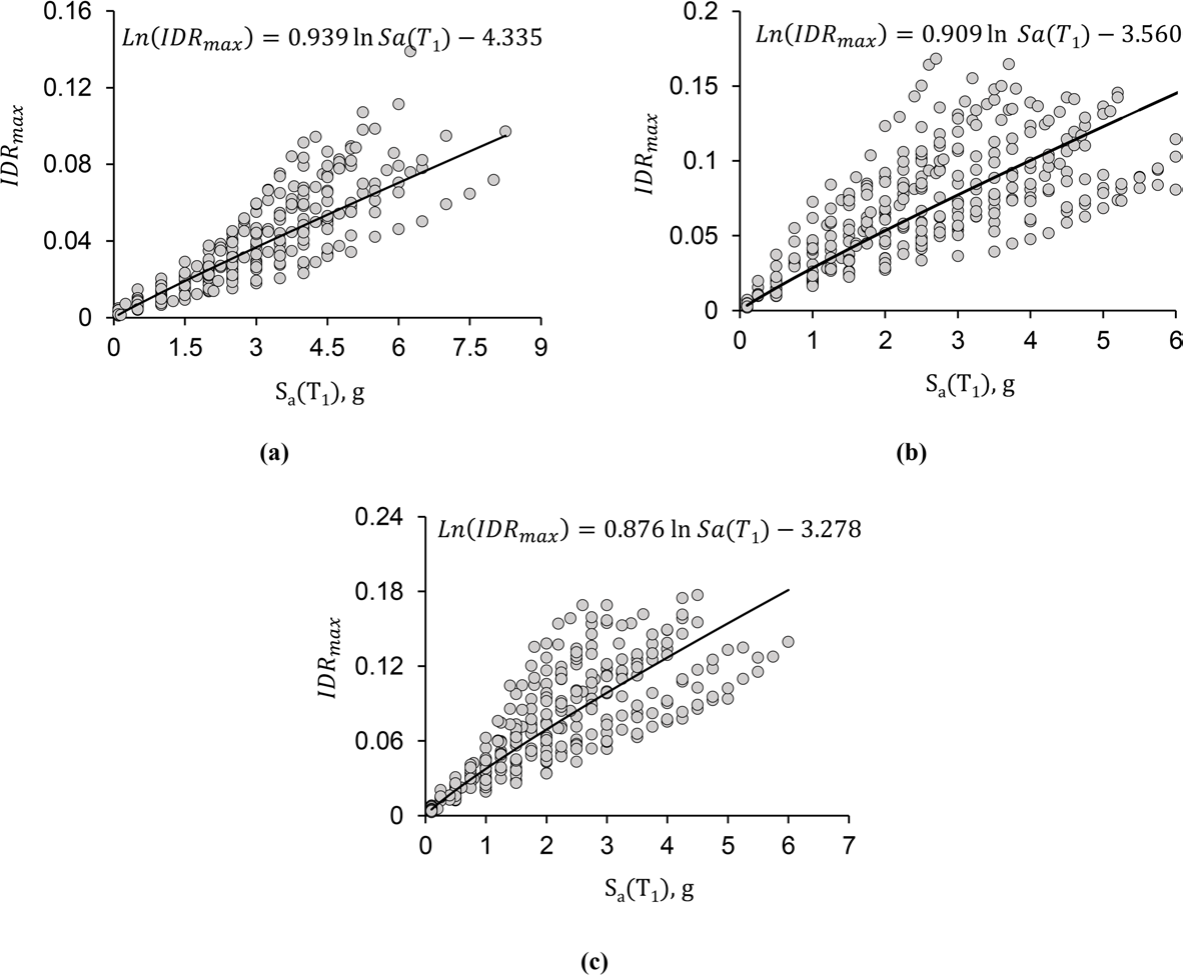
Incremental dynamic analysis results for special truss moment frames with 20-meter spans and special zone lengths of 1-meter: (a) 3-story frame, (b) 6-story frame, (c) 9-story frame.

To probabilistically evaluate the behavior of the structure prior to collapse, a statistical model is required to determine the relationship between the relevant data. In this study, the data include the intensity measure (*IM*) values and the seismic demand (*IDR*_max_), both obtained from incremental dynamic analysis (IDA). Figure 3 presents these data for the 3-, 6-, and 9-story STMFs with 20-meter spans and special zone lengths of 1 m as an example. Each point in Fig. 3 shows the seismic demand for a specific intensity measure, and all the data were obtained from time-history analyses using 22 different scaled ground motion records. The horizontal axis represents the intensity measure parameter S_*a*_(T_1_), while the vertical axis represents the seismic demand parameter *IDR*_max_ for the given frame.

Based on previous research in this field, the most appropriate mathematical form for describing the relationship between the intensity measure and the seismic demand parameter is a linear relationship between the natural logarithms of these two variables^27^. The general form of this relationship is as follows:

1 $$Ln\left( {ID{R_{max}}} \right)=aln{S_a}\left( {{T_1}} \right)+w+{\sigma _{NC}}\varepsilon$$

In this context, *a* and *w* are unknown parameters, and *σ*_NC_ is the model’s standard deviation for non-collapse data. The parameter *ε* is assumed to be a normally distributed random variable with a mean of zero and a standard deviation of one.

The Bayesian method was used to estimate the parameters of the seismic demand model. In the Bayesian statistical approach, in contrast to the classical method of statistics, where the mean values of the model parameters (here, *a* and *w*) are treated as fixed, the model parameters are considered random variables. In this approach, a probability distribution is used to model the associated uncertainty in the random variables^28^. Therefore, the parameters *a* and *w*, estimated using the Bayesian method, have ranges of possible values around their mean, and this uncertainty is incorporated into the model. The data used to estimate these model parameters are the same points obtained from the incremental dynamic analysis for the non-collapse dataset.

The use of the Bayesian method is effective in compensating for limited data, particularly in cases such as the present study, where large sample sizes are not available. Since it is often impossible to compute the posterior distribution analytically, Markov Chain Monte Carlo (MCMC) simulation methods are employed to generate samples from the posterior. These samples form a Markov chain whose stationary distribution is the desired posterior distribution. Through repeated sampling and discarding the initial (transient) iterations, the chain converges to a stable representation of the posterior, allowing for uncertainty in the model parameters to be accurately quantified^29^.

**Fig. 4:**
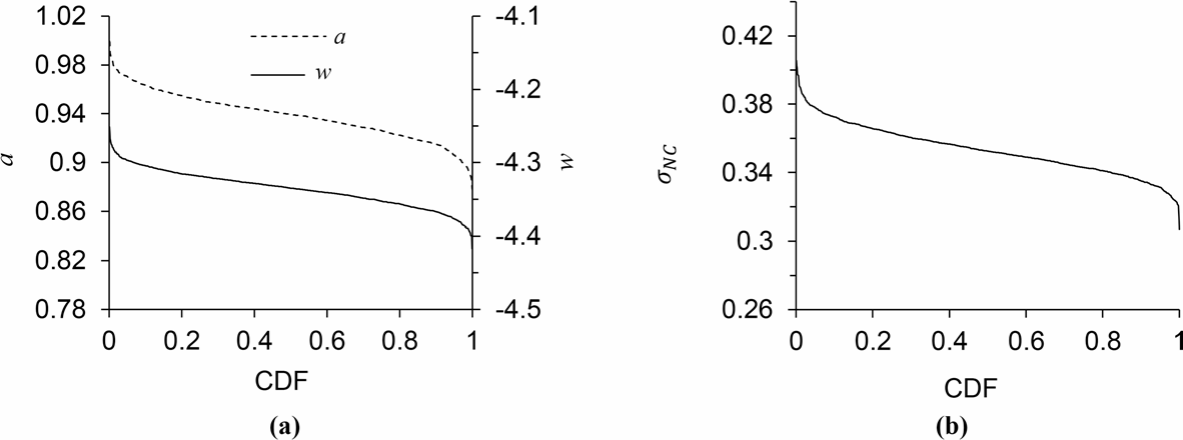
Probability distribution estimates of the seismic demand model parameters for the 3-story frame with 20-meter spans and 1-meter special zone lengths: (a) parameters *a* and *w*, and (b) parameter *σ*_NC_.

The simulation was implemented through the BayesX program^30^, to estimate the distributions of three unknown parameters in the seismic demand model: *a*, *w*, and *σ*_NC_​. The probability distribution curves for the 3-story STMF with 20-meter spans and special zone lengths of 1 m are shown as examples in Fig. 4. In these curves, the horizontal axis represents the cumulative probability. The estimated coefficients follow probability distributions centered around their mean values, with associated standard deviations that reflect their uncertainty. As shown in Fig. 4, the parameter *a* varies from 0.970 to 0.906 for the 3-story frame within the 0.05–0.95 probability range, while *w* and *σ*_NC_ vary from − 4.377 to -4.297 and 0.331 to 0.378, respectively.

The average values of the seismic demand model parameters (*a*, *w*, and *σ*_NC_) were calculated for all 27 STMFs examined. These parameters can be used to fit the best line to the non-collapse data according to Eq. (1). However, this line does not necessarily pass through all the data points; instead, it provides the best statistical fit and the highest consistency with the observed data. According to the results, the average value of parameter *a* ranges from 0.701 to 1.01, parameter *w* varies between − 4.33 and − 2.52, and parameter *σ*_NC_ ranges from 0.234 to 0.408. Results indicate that geometric characteristics of STMFs strongly affect the seismic demand model parameters *a* and *w*.

### Structural influence on seismic demand parameters

With the aim of determining a mathematical relationship between the seismic demand model parameters and the structural parameters, the impact of various factors on the seismic demand model was examined. For this purpose, the correlation coefficient was calculated to evaluate the strength of the relationship between each seismic demand parameter (*a* and *w*) and various structural variables. These variables include the period (T), span length (L), structure height (h), and special segment length (L_s_), along with all mathematical operations and combinations of these parameters. Based on the results, all independent structural parameters with a correlation coefficient greater than 0.5 with the seismic demand parameters were included in the mathematical model. To simplify the relationship, parameters with insignificant influence were excluded, resulting in Eq. (2), which provides a generalized form for predicting the values of parameters *a* and *w*:

2 $${Z_i}=c_{1}^{{\left( i \right)}}x+c_{2}^{{\left( i \right)}}ln\left( x \right)+c_{3}^{{\left( i \right)}}\sqrt x +c_{4}^{{\left( i \right)}}xy+c_{5}^{{\left( i \right)}}\sqrt {xy} +c_{6}^{{\left( i \right)}}\ln \left( {xy} \right)+c_{7}^{{\left( i \right)}}{{\mathrm{e}}^{xy}}$$

In this equation, *Z*_i_​ represents either *a* or *w*, depending on which parameter is being predicted. The appropriate form of the equation is used by specifying *i* = *a* when predicting *a* and *i* = *w* when predicting *w*. The coefficients $c_{i}^{{\left( a \right)}}$ and $c_{i}^{{\left( w \right)}}$ are constant values unique to each parameter. The variables *x* = *h/L* and *y* = *Ls/L* represent the height-to-span ratio and the normalized special segment length, respectively. After performing regression analysis on Eq. (2), the values of coefficients $c_{1}^{{\left( i \right)}}$ to $c_{7}^{{\left( i \right)}}$ were determined. These values are presented in Table 3.

**Table 3:** Regression-derived values of coefficients for Eq. (2).

Precited parameter	$c_{1}^{{\left( i \right)}}$	$c_{2}^{{\left( i \right)}}$	$c_{3}^{{\left( i \right)}}$	$c_{4}^{{\left( i \right)}}$	$c_{5}^{{\left( i \right)}}$	$c_{6}^{{\left( i \right)}}$	$c_{7}^{{\left( i \right)}}$
a	0.448	1.235	-3.052	-3.901	5.397	-0.613	0.677
*w*	-1.982	-4.845	13.213	14.013	-24.069	3.028	-1.627
${\mu _{\ln I{M^{cap}}}}$	-0.722	-1.999	4.927	3.672	-4.781	0.331	-0.832
$\ln {{\mathrm{P}}_C}$	24.301	63.955	-163.532	-107.416	218.408	-23.611	7.597

Normalized root mean square error, *e*_*nrms*_, was used to evaluate the accuracy and reliability of Eq. (2). This parameter is defined as the square root of the mean squared error between predicted and observed values, divided by the average of the observed values. The 6.2% normalized root mean square error for parameter *a* and the 3.9% normalized root mean square error for parameter *w* indicate that Eq. (2) estimates these parameters in this study with reasonable reliability and accuracy.

**Fig. 5:**
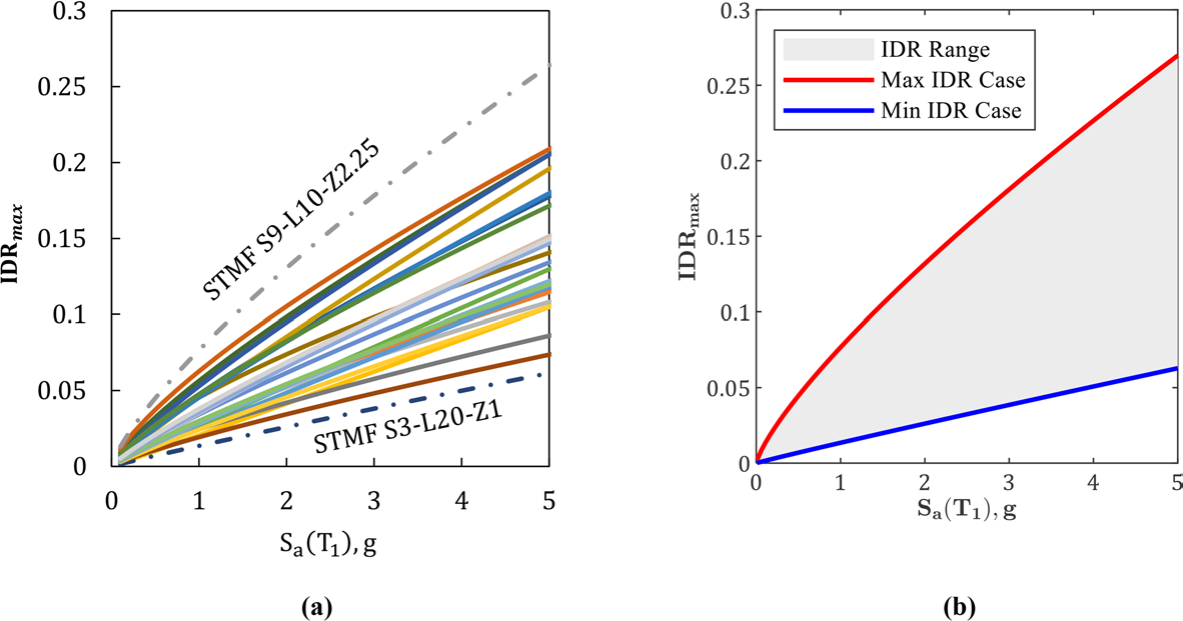
Seismic demand models for various configurations: (a) observed results for different STMFs, (b) predicted seismic demand based on Eq. (2).

Based on the seismic demand model in Eq. (1) and the estimated parameters from Eq. (2), the maximum interstory drift ratio (*IDR*_max_) was computed for various combinations of geometric parameters by varying the structure height (h), span length (L), and special segment length (L_s_) within the defined study range. Figure 5 illustrates the relationship between maximum interstory drift ratio (*IDR*_max_) and spectral acceleration at the first-mode period, S*a*(T_1_), for various configurations. It includes observed seismic demands for 27 STMFs (Fig. 5a) and the predicted seismic demand range based on Eq. (2) (Fig. 5b), shown as an envelope capturing the minimum, maximum, and seismic demand models variation within the geometric bounds of the considered frames. The minimum *IDR*_max_ in Fig. 5b corresponds to the dimensionless parameters *x* = 0.9 and *y* = 0.05, indicating a 3-story STMF with 20-meter spans and 1-meter special segments. The peak *IDR*_max_ in this figure occurs at x = 5.4 and y = 0.225, corresponding to a 9-story STMF with a 10-meter span and 2.25-meter special segment. Similarly, in Fig. 5a, the minimum and maximum observed seismic demands occur for S3-L20-Z1 and S9-L20-Z2.25, confirming the reliability of the proposed prediction model.

Additionally, Eq. (2) suggests that, across different story numbers, configurations with L = 20 m and L_s_ = 1.0 m result in lower seismic demands. It also shows that the expected seismic demand can be reduced by minimizing the height-to-span ratio (h/L) and the normalized special segment length (L_s_/L), providing a useful guideline for preliminary design. Furthermore, the analysis reveals that tall-slender frames with larger h/L ratios exhibit higher sensitivity of interstory drift demands to variations in Lₛ/L, while short-stiff frames with lower h/L show much smaller sensitivity.

The influence of a decreasing L_s_/L ratio on reducing seismic demand is related to the truss geometry. Decreasing the L_s_/L ratio and keeping the total span length constant increases the length of the portion of the truss outside the special segment. This portion, which contains the diagonal members, increases the overall frame stiffness. At the same time, a smaller L_s_ requires larger rotations in the special segment to accommodate a given interstory drift. As a result, the segment enters the inelastic range and dissipates energy at smaller drifts.

### Development of collapse fragility curves

**Fig. 6:**
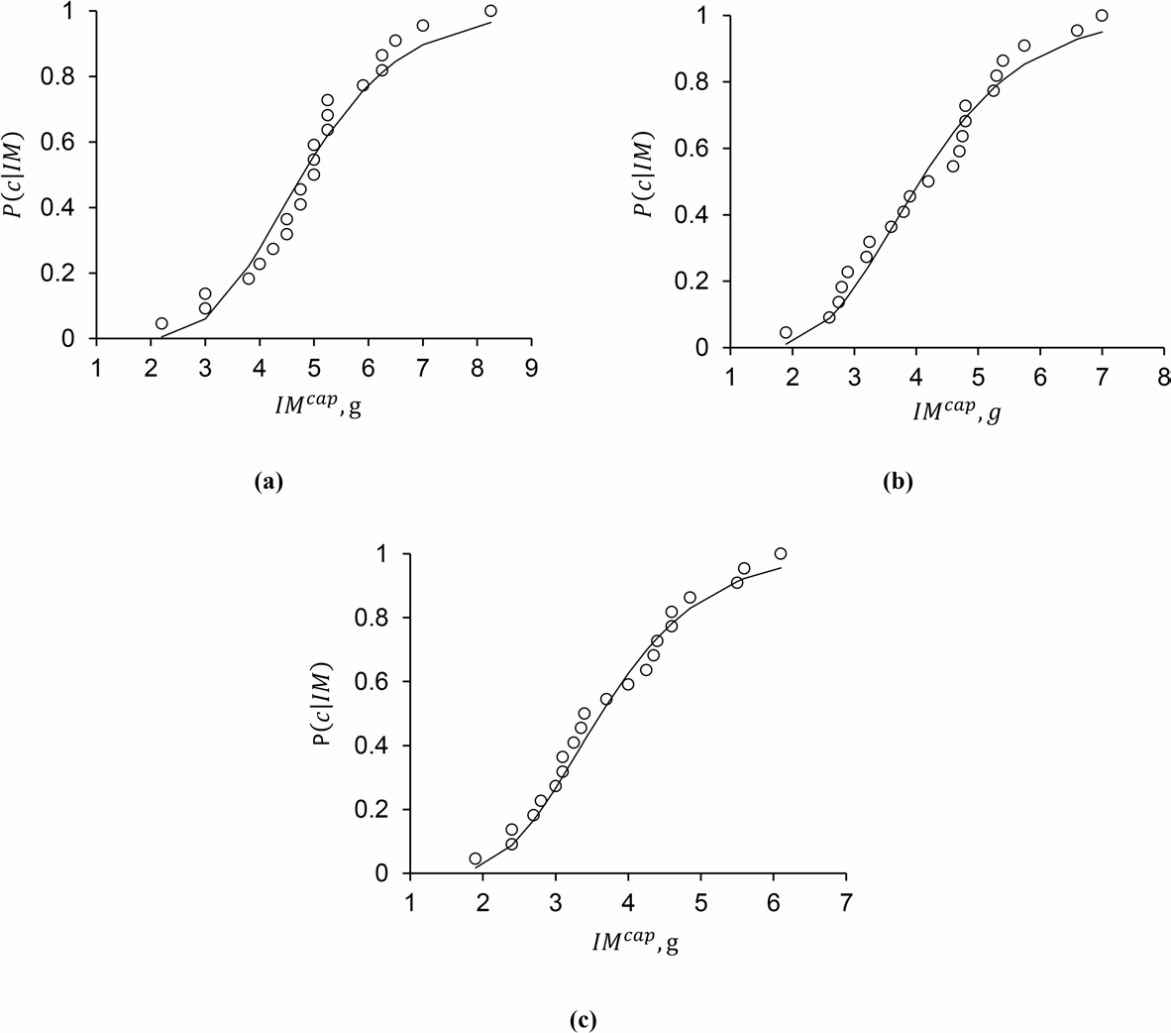
Collapse fragility curves for STMFs with 20-meter spans and 1-meter special segment lengths: (a) 3-story frame, (b) 6-story frame, (c) 9-story frame.

The IM corresponding to the collapse state in the structure for each record is usually referred to as the collapse capacity of the structure under that specific record (*IM*^*cap*^). The collapse capacity of a structure under different records varies and is expressed based on the intensity measure (*IM*) used for scaling the records. Figure 6 provides the collapse fragility curves for 3-, 6-, and 9-story frames with 20-meter spans and special segment lengths of 1 m for examples. The horizontal axis indicates the intensity measure (*IM*) values, and the vertical axis shows the probability of collapse, *P*(*C | IM*). The dots represent the collapse probability using the empirical distribution under the 22 records, and the continuous curve represents the fitted lognormal fragility function in terms of the intensity measure S_*a*_(T_1_) for the studied frames.

As discussed previously in section “Incremental dynamic analysis of STMFs”, collapse in this study is defined based on structure-specific performance criteria, namely numerical instability or a 20% reduction in the IDA slope. This definition differs from commonly used fixed drift-based collapse criteria in guidelines such as HAZUS^31^. Therefore, the resulting collapse fragility curves correspond to higher collapse drift ratios compared to common criteria, reflecting the ductile behavior of the studied frames. This ductility is directly related to the adopted design approach, in which inelastic action is intentionally concentrated within the special truss zone while columns and surrounding members remain almost elastic. Plastic hinges are formed in a distributed manner along the height of the frame, rather than being localized at a single story. The use of compact, ductile sections within the special zone enables the development of wide and stable hysteresis loops with significant energy dissipation capacity. This capability allows the frames to sustain significant seismic demands prior to collapse.

The consideration of the collapse fragility curves in Fig. 6 shows that, as the number of stories increases, the probability of collapse for a given intensity also increases. This figure also indicates that the lognormal distribution accurately describes collapse fragility curves and fits the observed data closely. The lognormal distribution is characterized by two parameters: the mean and standard deviation of the natural logarithm of collapse capacities, denoted by ${\mu _{\ln I{M^{cap}}}}$ and ${\sigma _{\ln I{M^{cap}}}}$ respectively. The parameter ${\sigma _{\ln I{M^{cap}}}}$ varies between 0.264 and 0.398, with a mean of 0.317, and the parameter ${\mu _{\ln I{M^{cap}}}}$ ranges between 0.659 and 1.566 depending on geometric characteristics of all 27 STMFs.

**Fig. 7:**
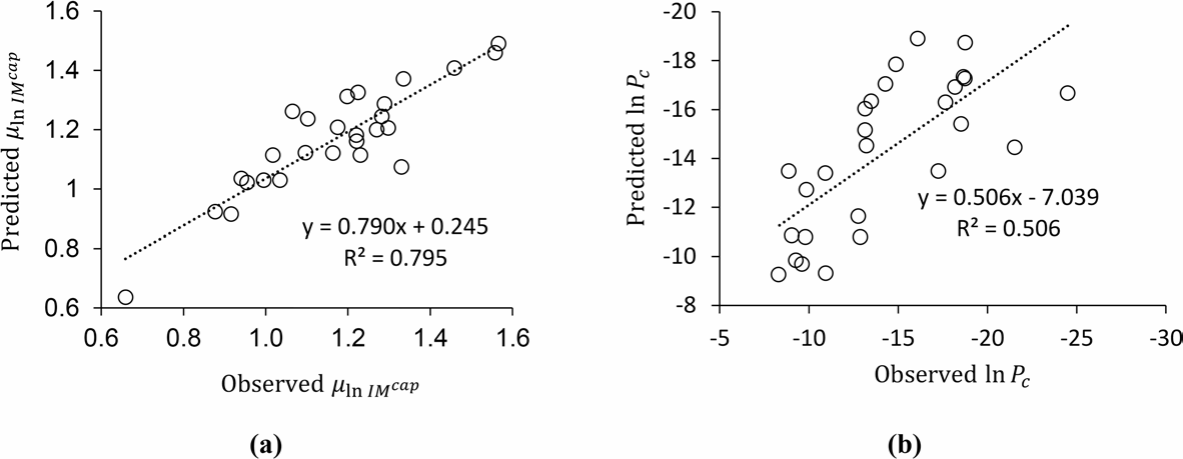
Observed versus predicted values for collapse assessment of the 27 STMFs: (a) median collapse capacity $\left( {{\mu _{\ln I{M^{cap}}}}} \right)$, (b) collapse probability in logarithmic scale $(ln{P_c})$.

A predictive model was developed for the mean of the natural logarithm of collapse capacities (${\mu _{\ln I{M^{cap}}}}$) based on the structural properties, similar to what was done previously for the seismic demand parameters in the non-collapse data. This approach enables the derivation of lognormal collapse fragility curves without performing incremental dynamic analyses. Accordingly, this parameter for special truss moment frames can be predicted using Eq. (2) with the specific coefficients given in Table 3. Figure 7a provides predicted versus observed values for median collapse capacity for 27 STMFS. As can be seen in this figure, the coefficient of determination (R^2^) is 0.795, showing that 79.5% of the variation in the observed data is captured by the model. This conclusion is also supported by the comparison graph, where the fitted line is close to the ideal y = x line and most points lie near it. Also, the normalized root mean square error, e_nrms_, was calculated and found to be 7.9%, indicating low dispersion in the predicted ${\mu _{\ln I{M^{cap}}}}$.

The optimization of the median collapse capacity highlights its dependence on key frame proportioning parameters. According to Eq. (2), a maximum capacity of 4.435 g was obtained at h/L = 0.9 and L_s_/L = 0.05, corresponding to minimal frame height and special segment length with maximal span length. Sensitivity analysis further shows that collapse capacity decreases with increases in both ratios. Within the considered range, the results confirm that maximizing collapse capacity is achieved by minimizing the frame’s aspect ratio (h/L) and the normalized length of its special segments (L_s_/L).

## Probabilistic seismic demand analysis of STMFs

**Fig. 8:**
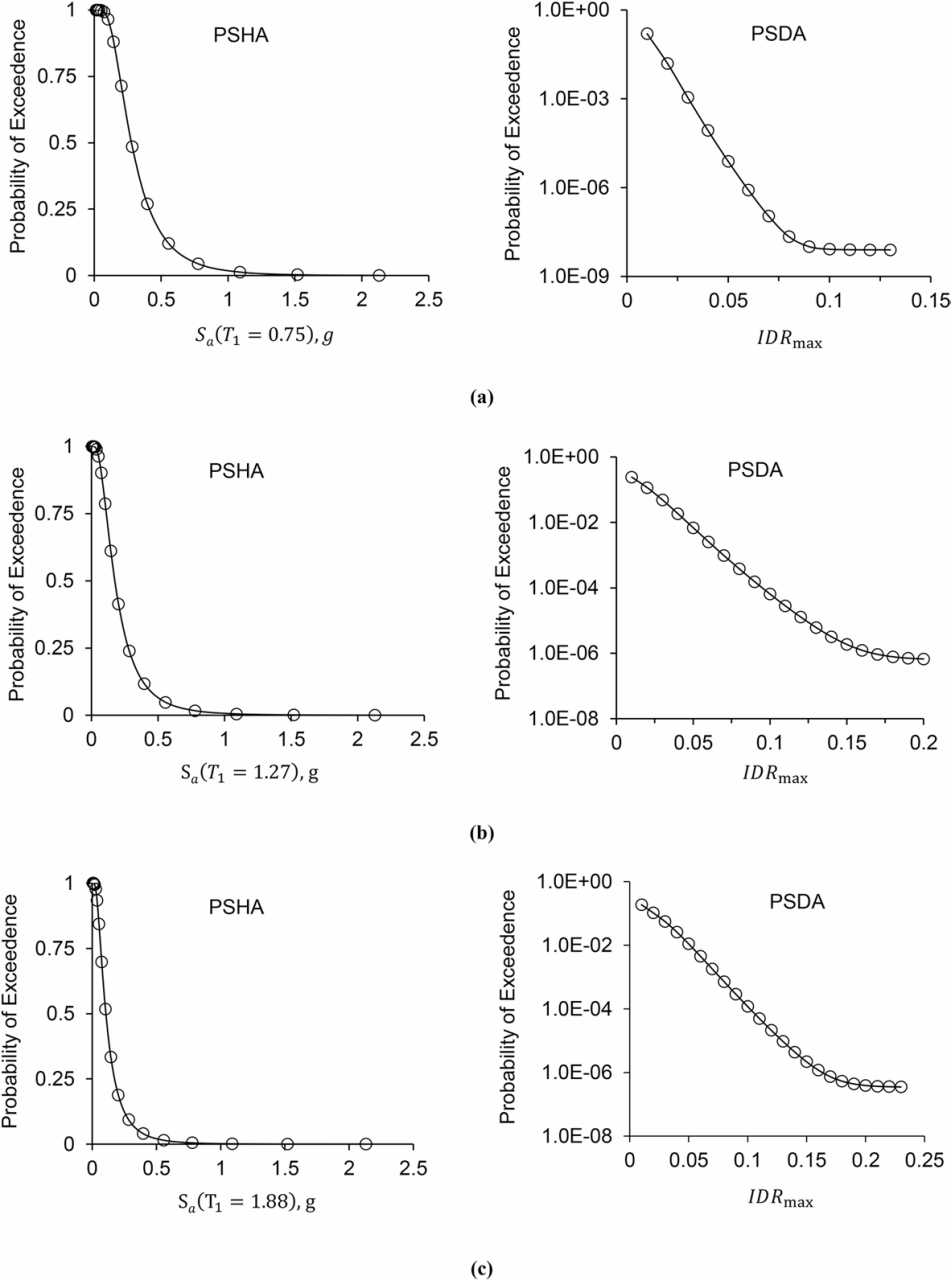
Seismic hazard curves and drift hazard curves over 50 years for frames with 20-meter spans and special segment lengths of 1-meter: (a) 3-story frame, (b) 6-story frame, (c) 9-story frame.

The final stage in the probabilistic seismic demand analysis is the plotting of drift hazard curves for the frames. To draw these curves, the results obtained from incremental dynamic analysis in both collapse and non-collapse states must be combined with the results of probabilistic seismic hazard analysis at the location where the structure is situated. For this study, the buildings are assumed to be located in Bojnord, a city in northeastern Iran. The left panels of Fig. 8a and c display PSHA curves for the S3-L20-Z1, S6-L20-Z1, and S9-L20-Z1, respectively, based on previously reported PSHA results^32^ and supplemented with complete site-specific data from the research project^33^. In these figures, the horizontal axis shows the intensity measure, represented by spectral acceleration at the first mode period, S_*a*_(T_1_), while the vertical axis shows the probability of exceeding a given earthquake intensity. The right panels of Fig. 8a and c display the drift hazard curves for STMFs. The horizontal axis corresponds to the maximum inter-story drift ratio (*IDR*_max_), and the vertical axis represents the annual probability of exceeding a specified seismic demand level (POE) over the structure’s design life of 50 years. By examining the seismic hazard curves in Fig. 8, it is observed that with an increase in the number of stories, the probability of exceeding a given level of seismic demand also increases.

Although drift hazard curves were calculated for Bojnord, it should be noted that predicting the probability of exceedance (POE) directly through structural parameters is more complex than in previous models. Unlike the seismic demand or collapse capacity parameters, POE depends not only on the structural characteristics but also on the site-specific seismic hazard, which varies from one location to another. Therefore, creating a general formula to estimate POE solely based on structural features is challenging. In contrast, collapse probability is more straightforward to calculate because it can be obtained by combining the collapse fragility functions, defining the probability of exceeding collapse, and seismic hazard results.

To develop an equation that predicts the logarithm of the collapse probability over a 50-year service life of STMFs, ${\mathrm{ln}P}_{c}$, without performing incremental dynamic analyses, a similar approach to that used in the previous section was applied. First, the collapse probability was obtained by combining the collapse fragility curves with the PSHA results for the city of Bojnord. Then, the coefficients of the predictive model in Eq. (2) were determined to estimate the natural logarithm of collapse probability directly. The values of the coefficients $c_{1}^{{\left( {\ln {P_c}} \right)}}$ to $c_{7}^{{\left( {\ln {P_c}} \right)}}$ are provided in Table 3. As shown in Fig. 7b, the predicted values of $\ln {P_c}$ are plotted against the corresponding observed values. It can be seen that the slope of the fitted line deviates from the ideal value of unity, and the coefficient of determination (R²) decreases to about 0.506. This represents a clear reduction in predictive accuracy compared to Fig. 7a, which corresponds to the prediction of the median collapse capacity. The normalized root mean square error (e_nrms_) was calculated as 20.9%, indicating that the model’s prediction errors correspond to 20.9% of the observed data variability. This level of error occurs when predicting the natural logarithm of collapse probability, which inherently leads to a larger discrepancy in the prediction of the collapse probability itself.

This result indicates that the proposed model is less accurate in estimating collapse probability compared to the seismic demand and fragility curve parameters. This reduced accuracy is expected, since collapse probability depends not only on structural features but also strongly on site-specific seismic hazard characteristics, which are not directly included in the model. Consequently, the model is not directly transferable to other regions without incorporating location-specific hazard parameters. Nevertheless, the proposed equation is intended to provide a preliminary estimate of $\ln {P_c}$ at early stages of assessment, prior to performing full IDA.

The collapse probability (*P*_c_) of STMFs under different geometric parameters was evaluated using Eq. (2). It was found that as the parameter of *x* (h/L) moves toward 0.9, the collapse probability decreases. The lowest collapse probability occurs at x = 0.9, which corresponds to the 3-story STMF with 20-meter spans. On the other hand, the upper boundary of y (Lₛ/L = 0.225), which represents the frames with 10-meter spans and 2.25-meter special segments, has the highest probability of collapse.

## Validation of proposed relationships

To further evaluate the accuracy of the proposed predictive models, a probabilistic seismic demand analysis was carried out on a 4-story STMF with a total height of 24 m, three identical spans of 12.5 m each, and a special segment length of 1.25 m. This frame was selected as an independent case study and was not part of the 27 STMFs used to derive the predictive relationships; therefore, it can be used to validate the proposed models without bias. The procedure followed for this validation example was similar to that applied to the 27 previously studied frames.

**Fig. 9:**
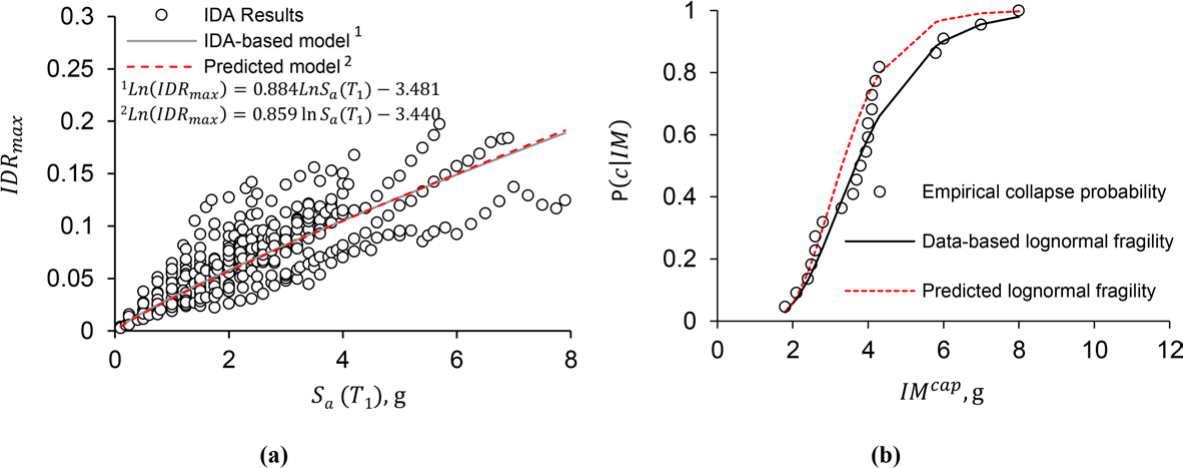
Incremental dynamic analysis results and collapse fragility curves for the 4-story special truss moment frame (STMF) with 12.5-meter spans and 1.25-meter special segment lengths: (a) IDA results, and (b) collapse fragility curves.

As in the previous analyses, the results of incremental dynamic analyses were classified into non-collapse and collapse categories. Figure 9 is presented in two parts to illustrate both aspects of the response behavior of the validation frame. Figure 9a and b provide the non-collapse IDA results and the corresponding collapse fragility curve for the 4-story frame.

The seismic demand parameters (i.e., parameters *a* and *w*) were estimated using the non-collapse data based on the Bayesian statistical method. In Fig. 9a, the gray line was plotted using the actual parameters (*a* = 0.859, *w* = -3.440), and the red dashed line represents the predicted parameters from Eq. (2) (*a* = 0.884, *w* = -3.481). There is very little difference between the predicted and actual values, as evidenced by the nearly identical slopes of these two lines. The relative errors for parameters *a* and *w* are approximately 2.9% and 1.2%, respectively, showing that Eq. (2) can be trusted to estimate the parameters of the seismic demand model accurately.

The fragility curve, predicted using Eq. (2), was shown in Fig. 9b with the dashed line, while the solid line shows the fragility curve based on the lognormal distribution fitted to the IDA results. The discrete points represent the collapse probabilities calculated from the empirical data under the 22 ground motion records. The difference between the data-based and predicted lognormal fragility curves is approximately 20%. As shown in the figure, the predicted curve reasonably represents the results from the detailed analysis, indicating that the suggested relationships can effectively represent the collapse behavior of STMFs and are helpful in creating fragility curves without needing a complete IDA. To further assess the predictive model’s accuracy in estimating collapse behavior, the median collapse capacity of the 4-story frame, calculated from Eq. (2) as 3.30 g, is compared to the value from the IDA results, which is 3.67 g. A relative error of 10.1% indicates that the proposed model estimates the median collapse capacity well.

These findings support the applicability of the predictive demand model for practical use. Overall, this validation case shows that predictive models work and provide valuable estimations of seismic demand behavior with relatively minimal computational effort. They are a practical choice compared to detailed nonlinear dynamic analyses, especially during early-stage design or screening-level assessments.

Lastly, to evaluate the model’s effectiveness in predicting collapse probability, this probability was calculated by combining the results of the collapse fragility curve with the seismic hazard curve for the location. According to Eq. (2), the predicted probability of collapse is 3.47 × 10^− 7^, while the actual value, derived from the numerical integration of the fragility curve and the PSHA results, is 1.63 × 10^− 6^. This deviation corresponds to a relative error of about 79%, indicating limited accuracy in directly predicting collapse probability. As previously discussed, direct collapse probability predictions show limited accuracy due to dependence on site-specific hazard, as reflected in the previous metrics (R^2^, e_nrms_). Nevertheless, as shown in Figs. 7a and 9b, the model demonstrates good accuracy in predicting collapse fragility curves of STMFs, even though its performance in direct collapse probability estimation remains limited.

Building on the previous validation for low-rise frames, the predictive capability of the proposed models was further evaluated for high-rise frames. For this purpose, one of the nine-story STMFs from the dataset was excluded and treated as an independent validation case. The predictive models were recalibrated using the remaining 26 frames, and the resulting coefficients were used to estimate the seismic demand and collapse fragility of the excluded frame. The comparison shows that the predicted response closely matches the IDA-based results. This confirms that the proposed models can accurately predict both seismic demand and collapse fragility for high-rise frames not included in the original dataset.

## Conclusion

Probabilistic seismic demand analysis (PSDA) was conducted to investigate the seismic response of 27 three-span STMFs with different numbers of stories, span lengths, and special truss segment lengths. The main conclusions of this study are as follows:

- Results from incremental dynamic analyses were used to develop predictive models for seismic demands, collapse fragility curves, and collapse probabilities. Among the investigated variables, the height-to-span ratio (h/L) and the special segment length-to-span ratio (Lₛ/L) have the greatest influence on seismic response, so prediction equations were developed in terms of h/L and Lₛ/L.
- Minimizing the height-to-span ratio (h/L) and the normalized special segment length (Lₛ/L) reduces non-collapse seismic demand while simultaneously increasing the median collapse capacity (${\mu _{\ln I{M^{cap}}}}$). This demonstrates that the same geometric configurations that reduce non-collapse seismic demand also enhance collapse resistance.
- The average collapse drift (${\overline {{IDR}} _{max,c}}$) of STMFs ranges between 0.075 and 0.20 and was estimated with reasonable accuracy as a function of the fundamental period of the STMF using a power law.
- Drift hazard curves demonstrate that as the number of stories increases, the probability of exceeding a given level of seismic demand also increases.
- The independent validation using a 4-story frame confirmed the generalizability of all proposed predictive equations. This supports using the predictive equations in early-stage design and risk assessment without the need for full nonlinear time-history analysis.
- At the collapse level, the proposed model is suitable for preliminary assessment of collapse probability and for accurate estimation of collapse fragility curves of STMFs. Its accuracy in directly predicting collapse probability is limited mainly because site-specific seismic hazard parameters are not included in the model.
- The predictive model is limited to the range of structural configurations investigated in this study. This range includes three-span STMFs with a single special truss segment at the mid-span. Specifically, the model applies to frames with three to nine stories, a height-to-span ratio (h/L) between 0.90 and 5.40 and a ductile segment-to-span length ratio (L_s_/L) between 0.05 and 0.225. Application of the proposed equations outside these geometric and structural ranges should be treated with caution. It should be noted that these coefficients are valid for frames designed according to AISC 341 − 16 seismic provisions for high seismicity regions.
- Future studies could extend the applicability by examining different numbers of spans, more than one specific truss segment, alternative geometric configurations, and near-faults seismicity. In addition, future versions of the model should include site-specific seismic hazard parameters to improve collapse probability prediction.

## Data Availability

The datasets generated and analyzed during the current study are available from the corresponding author on reasonable request.
